# Examining warfarin underutilization rates in patients with atrial fibrillation: Detailed chart review essential to capture contraindications to warfarin therapy

**DOI:** 10.1186/1477-9560-6-6

**Published:** 2008-06-03

**Authors:** Ajay Srivastava, Michael Hudson, Ihab Hamoud, Joao Cavalcante, Chetan Pai, Scott Kaatz

**Affiliations:** 1 Department of Internal Medicine, Henry Ford Hospital, Detroit, MI, USA; 2 Heart and Vascular Institute, Henry Ford Hospital, Detroit, MI, USA

## Abstract

**Introduction:**

Atrial fibrillation affects an estimated 2.5 million Americans and incurs an average annual stroke risk of 4.5% per year. Despite warfarin reducing stroke risk by approximately 66%, prior studies show warfarin usage rates to be about 50%. However, the methods that define warfarin as "inappropriate underutilization" might not be sensitive enough to pick up relative contraindications. We assessed the inappropriate underutilization of warfarin in atrial fibrillation patients at our hospital by abstracting individual patient charts.

**Methods:**

Medical records were reviewed to determine stroke risk factors, warfarin use, and documented contraindications to warfarin use in 364 consecutive patients with atrial fibrillation.

**Results:**

Amongst 364 atrial fibrillation patients, 54.6% received warfarin anticoagulation. Overall, 29.5 % of patients had documented reasons for not prescribing warfarin. Primary reasons listed by treating physicians included: gastrointestinal bleed 10.7%, secondary/transient atrial fibrillation 8.2%, and fall risk 6.3%. Only 7.1% of the patients had no documented reasons for the lack of warfarin use.

**Conclusion:**

Consistent with previous reports, 45.4% of patients in this atrial fibrillation cohort were not prescribed warfarin. However, after reviewing medical charts for documented reasons why warfarin was not used, the inappropriate underutilization rate was only 7.1%. These findings suggest that studies utilizing administrative database and ICD-9 CM coding might overestimate warfarin underutilization.

## Introduction

Atrial fibrillation (AF) currently affects an estimated 2.5 million patients in the United States and this number is expected to double within the next 20 years. [1,2] AF is particularly prevalent in the elderly population affecting 1 in 10 people over the age of 80 and accounting for one quarter of all strokes in the elderly population. [1] Patients with AF not receiving antithrombotic therapy have a stroke risk of 4.5% per year. [3-5] While studies have shown that warfarin therapy could reduce this stoke risk by about 66%[6], it is distressing to note that warfarin therapy remains widely underutilized [7-9], with bleeding risk and monitoring concerns being cited as the most common reasons for nonuse.

Prior studies analyzing warfarin utilization in patients with AF have generally relied on administrative data in determining patient's candidacy for warfarin therapy. [7-12] Due to the inherent study design and methodology, warfarin underutilization rates can be overestimated in such studies by including patients with relative or perceived contraindications to anticoagulation. The objectives of this study were a) To determine the rate of warfarin underutilization in a cohort of AF patients by retrospectively reviewing medical charts including physician verification of electrocardiograms (EKG), b) To compare methodologies of prior studies investigating warfarin underutilization and the role of chart review in estimating warfarin underutilization in AF patients and c) Attempt to identify physician-professed reasoning for not initiating warfarin therapy and analyze the adequacy of these reasons/contraindications.

## Methods

### Study Population

We retrospectively reviewed medical records of 364 patients with atrial fibrillation, after EKG confirmation by a cardiologist, and at least 1 year of follow-up from the index EKG. Index EKGs were obtained from inpatient and outpatient data from January 1, 2002, through December 31, 2003. The study was approved by the Henry Ford Hospital Institutional Review Board.

### Variables

Electronic medical records were reviewed for stroke risk factors, which included baseline demographic variables and previous diagnoses of stroke/transient ischemic attack (TIA), congestive heart failure (CHF), myocardial infarction (MI), coronary artery disease (CAD), hypertension, and diabetes mellitus (DM). Clinic notes and hospital discharge summaries were reviewed to identify physician documented reasons for not prescribing warfarin therapy. Patient race/ethnicity was obtained from demographic profiles listed in electronic medical records. Gastrointestinal bleed was considered to be a limiting factor if there was a physician note documenting prior history of bleeding or guaiac-positive stools. Non-gastrointestinal bleeds included bleeding from all other causes, such as hematoma and trauma. Fall risk was recorded if there was a physician note stating increased risk for falls or unfavorable fall risk to warfarin benefit ratio. Patients were considered to have transient or secondary AF if there were EKGs documenting AF during an acute event, such as surgery or acute medical illness with all subsequent EKGs showing normal sinus rhythm and physician notes stating warfarin was not indicated. Patients with a prior diagnosis of AF that had converted to sinus rhythm during follow-up were also included in this group. Patient compliance was considered a factor if there were physician notes stating that the patient had a previous history of non-compliance to medical therapy. Coagulopathy included patients with an underlying bleeding or coagulation disorder placing them at an increased risk for bleeding. CHADS_2 _(C-cardiac failure, H-hypertension, A-age > 75 years, D- diabetes mellitus, S- stroke) index is a point-based system to stratify stroke risk and thereby help guide with anti-coagulation decision-making in patients with AF.[3] 2 points are assigned for previous stroke or TIA and 1 point each for age > 75 years, hypertension, diabetes mellitus or recent congestive heart failure. The total score calculated then helps determine the stroke risk, with higher the CHADS_2 _score greater the risk of stroke.

### Statistical Analysis

A descriptive analysis was performed to compare baseline clinical and demographic characteristics between patient groups receiving warfarin and those not receiving warfarin. The chi-square test was used for categorical variables and Student's *t *test for the continuous variables. The CHADS_2 _score between the two groups were compared using the Wilcoxon rank sum test. After breaking down the CHADS_2 _variable into its individual scores, a Cochran-Armitage trend test was performed to compare warfarin usage based on CHADS_2 _score, with a *p *value of .05 suggesting a statistically significant difference. All statistical analyses were performed using SAS software (version 8.2).

### Comparing Prior Studies Investigating Warfarin Underutilization

A systematic literature search using the MEDLINE computerized database was performed (January 1, 1995, to December 1, 2005). Relevant studies were identified by using the following keywords: "*warfarin, underutilization, coumadin, fall risk, atrial fibrillation, CHADS*_2_*, anti-coagulation in atrial fibrillation, stroke risk, gastrointestinal bleed, transient atrial fibrillation, intracranial hemorrhage, elderly*". Bibliographies of each article were reviewed to identify additional articles. Articles relevant to the present study were than tabulated into a table and warfarin utilization rates were compared based on study period, health care setting and study methodology.

## Results

Of the 364 patients, 199 patients (54.6 %) received warfarin. Patients who received warfarin were younger than 75 years compared to patients who did not receive warfarin (table 1), and African American patients were less likely to receive warfarin. Only 5 patients (1.3%) had a CHADS_2 _score of zero and 34 patients (9.3%) had a CHADS_2 _score of 1, suggesting a moderate-to-high risk of thromboembolism in the study population. There were no significant differences in the proportion of patients with prior hypertension, stroke, cardiac disease, or diabetes between patients prescribed warfarin and those not receiving anticoagulation. As shown in Table 1, the mean overall CHADS_2 _score was higher in patients not receiving warfarin compared to patients on warfarin therapy (3.2 ± 1.3 vs. 2.8 ± 1.3, *p *< .004). Also, there were more patients with a CHADS_2 _score of 3 or greater in the cohort not on warfarin therapy, compared to the group on warfarin therapy (70% vs. 56%, p= 0.004). A detailed review of medical records revealed documented reasons for not prescribing warfarin in 83% (137/165) of patients not receiving warfarin. Prior or recent gastrointestinal bleeding was the most often cited contraindication to warfarin therapy; with other perceived reasons listed in Table 2. Only 7.1% (26/364) patients did not have a documented reason for warfarin non-use.

**Table 1:** Characteristics of Patients with AF classified based on Warfarin Use

**Characteristics**	**Received Warfarin n = 199**	**Did Not Receive Warfarin n = 165**	***p *value**
**Age, years, mean**	**76.3 ± 11.3**	**81.3 ± 10.3**	< 0.001*
**Age 75 ≥ y, n (%)**	**125 (62.8)**	**134 (81.2)**	< 0.001*
**AA Race, n (%)**	**74 (37.2)**	**82 (49.7)**	0.016*
Women, n (%)	76 (38.2)	79 (47.9)	0.063
CAD, n (%)	92 (46.2)	87 (52.7)	0.217
**Mean CHADS_2 _Score ± S.D**	**2.8 ± 1.3**	**3.2 ± 1.3**	0.004*
**CHADS_2 _Score- 3 or greater (%)**	**111 (56)**	**116 (70)**	0.004*
**Chads_2 _Score**			
No. of points, n (%)			
0	3 (1.5)	2 (1)	
1	22 (11)	12 (7)	
**2**	**63 (32)**	**35 (21)**	
**3**	**59 (30)**	**58 (35)**	
4	32 (16)	28 (17)	
**5**	**15 (7.5)**	**22 (14)**	
**6**	**5 (2.5)**	**8 (5)**	
Heart Failure, n (%)	120 (60.3)	103 (62.4)	0.679
Diabetes, n (%)	68 (34.2)	66 (40.0)	0.251
Hypertension, n (%)	179 (90.0)	149 (90.3)	0.911
Prior Stroke, n (%)	47 (23.6)	43 (26.1)	0.591

Abbreviations: AA- African American, CAD- Coronary Artery Disease, CHADS2- C-cardiac failure, H-hypertension, A-age > 75 years, D- diabetes mellitus, S- stroke, S.D- Standard deviation

*Statistically significant, *p *< 0.05

**Table 2:** Physician's Perceived Reasons for Not Initiating Warfarin Therapy (N = 137)

1. History of Gastrointestinal Bleed	- 39 (28%)
2. Transient/Secondary AF	- 30 (22%)
3. Fall Risk	- 23 (17%)
4. Patient preference	- 19 (14%)
5. History of Non-Gastrointestinal Bleed	- 11 (8%)
6. Poor Patient Compliance	- 8 (6%)
7. Coagulopathy	- 5 (4%)
8. Miscellaneous	- 2 (1%)

Table 3 compares the prior studies that have examined warfarin utilization in AF patients. Warfarin utilization rates very between 38%–s61% in the larger population studies that employ ICD-9-CM (The International Classification of Diseases, Ninth Revision, Clinical Modification) diagnostic codes, administrative or billing databases for assessing warfarin use while the rates drop significantly lower in the smaller patient cohort studies that review medical charts and account for reasons for warfarin nonuse.

**Table 3:** Comparison of Patient Selection Methods in Prior Studies Examining Warfarin Utilization in AF Patients

**Study Period**	**Author**	**Setting**	**Patient Selection for AF based on**	**Patient Population Size**	**Warfarin Utilization %**	**"Warfarin Candidates" not on Warfarin %**	**Method of determining Warfarin Utilization**
1995–1998	Weisbord et al.^13^	VA	Medical Chart review	1289	65	3.5	Medical Chart reviewed by physicians for documented contraindication
1996–1997	Go et al.^11^	Large HMO	ICD-9-CM code	13428	55	37.9	Pharmacy database, Outpatient INR, ICD for "Coumadin Therapy"
1997–1998	McCormick et al.^12^	Long term Care Facility	Medical Chart reviewed for EKG diagnosis or documentation by physician	429	42	32	Warfarin prescriptions, INR, Physician Notes
2000–2002	Waldo et al.^9^	Teaching, community and VA hospitals	ICD-9-CM code	945	54	22	ICD-9 & Medical chart review
2000–2005	Darkow et al.^7^	HMO	ICD-9-CM code	12539	39	61	Unable to obtain
2001–2003	Hylek et al.^8^	Urban teaching hospital	Electronic medical chart review and ECG verified AF	405	51	2	Electronic medical chart review for physician cited reasons for warfain non-use
2004–2005	Our Study	Single Tertiary Care Hospital	Medical Chart review and physician confirmation of AF by EKG	364	54	7.1	Medical chart reviewed by physicians for documented contraindication

## Discussion

The present study conducted at a single tertiary care center shows that 45% of AF patients did not receive antithrombotic therapy with warfarin. However, individual medical chart review revealed that 83% of patients not prescribed warfarin had a real or perceived documented reason for warfarin nonuse cited by the healthcare provider. After taking these factors into account, only 7.1% of ideal candidates for warfarin therapy were not receiving warfarin therapy.

McCormick et al [12] showed an overall warfarin utilization of 42% (180/429), but a further detailed chart review of 83 "ideal warfarin candidates" demonstrated 75% warfarin use in those with no potential warfarin contraindications. Similarly, Weisbord et al [13] and Hylek et al [8] showed a dramatic decrease in warfarin underutilization rates after employing chart reviews and excluding patients with documented contraindications to warfarin therapy. Further, in a recent report Waldo et al [9] from the NABOR (National Anticoagulation Benchmark Outcomes Report) Committee studied warfarin underutilization in hospitalized patients with AF and showed initial warfarin underutilization rates of about 54.4%, but in the high-risk stroke AF patients, after taking bleeding considerations into account, the underutilization was reduced by 10%. Review of other prior studies examining AF underutilization (table 3) cumulatively suggest that published rates of warfarin underutilization are highly influenced by the rigor used to review patient charts, and that administrative database reviews can underestimate the proportion of patients with real or perceived relative contraindications to warfarin use.

As shown in table 1, a greater percentage of patients in the non warfarin group had a CHADS_2 _score of 3 or greater (70% vs. 56%, *p *= 0.004). This is of extreme concern as AF patients with high calculated stroke risk are not being treated with warfarin therapy due to one or more physician perceived contraindications. Primary documented reasons for warfarin non-use in our study included history of GI bleeds (39/165), transient/secondary AF (30/165), and fall risk (23/165).

History of GI bleed accounted for 29% (39/137) of the warfarin candidates not on warfarin therapy in our study. Our finding is in agreement with prior studies, that physicians are less likely to initiate antithrombotic therapy in patients with a history of GI bleed.[14,15] This concern is understandable; considering persons on warfarin therapy are not only more likely to develop major GI tract bleeding (2-fold higher) but also, this risk also increases with age. [16,17] Yet, Man-Son-Hing and Laupacis showed in their study [18] that in order not to benefit from warfarin therapy, the person must have a significantly high risk of upper GI tract bleeding (> 10.4% per year) or a stroke risk less than 2.4% per year (patients with AF carry a 4.5% annual stroke risk). They further suggested a treatment model to help guide antithrombotic therapy in AF patients with a history of upper GI tract bleeding. As lower GI tract bleeding was not included in the model, Beyth suggested in the same study, that a treatment model to help guide physicians should attempt to include other factors known to augment the risk for both upper and lower GI bleed such as older age, non-steroidal anti-inflammatory drugs, [19,20] steroid use, [21] proton pump inhibitors, alcohol consumption, cigarette smoking and warfarin noncompliance. GI bleed can occur due to different reasons and in some cases can be related to a specific causative factor or event that led to the bleed. It is reasonable to think that removal of the causative factor or treatment should not place the patient at risk for future bleeding or make them ineligible for warfarin therapy, especially if the therapy is to occur years later. Future research should examine this specific cohort closely to help better identify potential patients who could otherwise benefit from warfarin therapy.

The second most common reason for not starting warfarin therapy was transient or secondary AF. It is clear from the current literature that this group of patients should be treated as AF patients and be given warfarin therapy.[9,22] To correct this oversight and diminish the number of such cases, healthcare providers must be made aware of warfarin therapies and educated in depth.

Fall risk was the third most commonly reported reason in our patient cohort for not prescribing warfarin, and as falls are associated with the elderly population [23-25] in which AF is highly prevalent, this is particularly a noteworthy finding. There is much ambiguity involved when a patient is labeled as "fall risk". [26] As shown in prior studies, patients are often not started on anti-coagulation for fear of the bleeding risk that is associated with falls.[27] We agree that this is a difficult topic to assess, as fall or neurological illness histories not routinely investigated or taken into account, but such histories are highly variable and subjective, based on the healthcare provider and the patient. Despite all these issues, we believe this is a key area for clinical improvement. Previous studies have cited fall risk and intracranial hemorrhage as the major reasons for not initiating anticoagulation in AF patients, especially the elderly.[23,27,28] The recent Birmingham Atrial Fibrillation Treatment of the Aged (BAFTA) trial [25] showed that even in an older AF patient group, warfarin decreased fatal stroke (1.8% per yr vs. 3.8% per year), and did not increase intracranial hemorrhage risk (1.4% per year vs. 1.6% per year) compared to aspirin therapy. Moreover, Gage et al [24] demonstrated that while older patients are at high risk for falls and carry an increased risk for intracranial hemorrhage, these patients also have a higher stroke risk, and therefore would likely benefit from anticoagulant therapy. These studies emphasize the point that fear of intracranial hemorrhage might be overestimated in this patient cohort. Indeed, a recent study by Jacobs [29] showed that effective management of warfarin can be achieved in the elderly by careful attention to these issues. Healthcare institutions should also consider employing objective methods to identify patients at fall risk by utilizing screening forms and taking patient, physician, and social factors into consideration.

This study has limitations inherent to a retrospective chart review. Our study was confined to a single center, and practice patterns might vary at other hospitals. Data collection regarding warfarin contraindications was limited to the available medical record content and no pre-specified or verifiable criteria were applied to the physician-cited contraindications for nonprescription of warfarin. The limited sample size in the group with documented perceived contraindications prevented further analysis to examine the adequacy of the contraindication to warfarin therapy.

In conclusion, our study found documented reasons for warfarin nonuse in a majority of the patients, as after a detailed medical chart review, only 7.1% of ideal candidates for warfarin therapy were not receiving it. These findings suggest that administrative database studies might overestimate warfarin underutilization in AF patients and that physician omission/error is not the primary cause of warfarin underutilization. There is an urgent need for prospective studies to investigate relative warfarin efficacy and bleeding risks in patients with perceived warfarin contraindications, as it would help guide healthcare providers in warfarin prescribing and consequently reduce the risk of AF-related disabling strokes.

## Abbreviations

AF: atrial fibrillation; ICD- CM: The International Classification of Diseases, Ninth Revision, Clinical Modification; BAFTA: Birmingham Atrial Fibrillation Treatment of the Aged; CHF: congestive heart failure; CAD: coronary artery disease; DM: diabetes mellitus; MI: myocardial infarction; NABOR: National Anticoagulation Benchmark Outcomes Report; TIA: transient ischemic attack.

## Competing interests

The authors declare that they have no competing interests.
